# Experiences of parents of children with rare neurogenetic conditions during the COVID-19 pandemic: an interpretative phenomenological analysis

**DOI:** 10.1186/s40359-023-01205-3

**Published:** 2023-06-08

**Authors:** Jessica A. Martin, Kathryn Robertson, Caroline Richards, Gaia Scerif, Kate Baker, Charlotte Tye

**Affiliations:** 1 Department of Psychology, Institute of Psychiatry, Psychology and Neuroscience, King’s College, London, UK; 2 MRC Cognition and Brain Sciences Unit, University of Cambridge, Cambridge, UK; 3 Cerebra Centre for Neurodevelopmental Disorders, School of Psychology, University of Birmingham, Birmingham, UK; 4 Department of Experimental Psychology, University of Oxford, Oxford, UK

**Keywords:** Neurogenetic conditions, COVID-19, Mental health, Wellbeing, Interpretative phenomenological analysis

## Abstract

**Background:**

The Coronavirus disease 2019 (COVID-19) pandemic has impacted parental and child mental health and wellbeing in the UK. This study aimed to explore the experiences of parents of children with rare neurological and neurodevelopmental conditions with a known or suspected genetic cause (neurogenetic) across the first year of the pandemic in the UK.

**Methods:**

Semi-structured interviews were conducted with 11 parents of children with rare neurogenetic conditions. Parents were recruited via opportunity sampling from the CoIN Study, a longitudinal quantitative study exploring the impact of the pandemic on the mental health and wellbeing of families with rare neurogenetic conditions. Interviews were analysed using Interpretative Phenomenological Analysis.

**Results:**

Four main themes were identified: (1) “A varied impact on child wellbeing: from detrimental to ‘no big drama’”; (2) “Parental mental health and wellbeing: impact, changes, and coping”; (3) “'The world had shut its doors and that was that’: care and social services during the pandemic”; and (4) “Time and luck: abstract concepts central to parents’ perspectives of how they coped during the pandemic”. The majority of parents described experiencing an exacerbation of pre-pandemic challenges due to increased uncertainty and a lack of support, with a minority reporting positive effects of the pandemic on family wellbeing.

**Conclusions:**

These findings offer a unique insight into the experiences parents of children with rare neurogenetic conditions across the first year of the pandemic in the UK. They highlight that the experiences of parents were not pandemic-specific, and will continue to be highly relevant in a non-pandemic context. Future support should to be tailored to the needs of families and implemented across diverse future scenarios to promote coping and positive wellbeing.

**Supplementary Information:**

The online version contains supplementary material available at 10.1186/s40359-023-01205-3.

## Background

Since the onset of the first national lockdown in March 2020, the COVID-19 pandemic has drastically impacted all aspects of daily life in the UK [1]. Children with rare neurological and neurodevelopmental conditions with a known or suspected genetic cause (neurogenetic) and their parents are uniquely vulnerable to the stressors brought on by the pandemic due to pre-existing difficulties and heightened risk of poor mental health [2–5]. Therefore, there is a critical need to gather in-depth information about the experiences of families with rare neurogenetic conditions across different stages of the pandemic.

Recent quantitative research suggests that the first UK lockdown (March 2020–July 2020) negatively impacted the mental health and wellbeing of children with rare neurogenetic conditions [6, 7]. In these studies, children with rare neurogenetic conditions were reported to experience behavioural difficulties, stress navigating social restrictions and isolation, and worries surrounding COVID-19 infection [6, 7]. Moreover, the severity of internalising and externalising behavioural difficulties, and their impact on daily functioning were found to be significantly greater in children with rare neurogenetic conditions than children with special educational needs and disability or neurodevelopmental disorders (SEND/ND), and children in the wider population. Further, the magnitude of these differences were greater than those reported in independent studies prior to the pandemic [6]. Taken together these findings highlight the negative impact of the pandemic on the mental health and wellbeing of children with rare neurogenetic conditions.

Contrastingly, the impact of the pandemic on the mental health and wellbeing of parents of children with rare neurogenetic conditions is less clear. During the first UK lockdown, a notable proportion of parents of children with rare neurogenetic conditions experienced borderline or abnormal levels of anxious (46%) and depressive (26%) symptoms [7]. Overall the severity of these symptoms and stress were found to be similar to parents of children in the wider population when controlling for demographic variables and isolation status [6]. Moreover, the magnitude of differences between parent groups was the same or smaller than reported pre-pandemic differences [6]. Together these preliminary findings imply parents of children with rare neurogenetic conditions experienced poor mental health during the first UK lockdown, yet this was no worse than parents in the wider population and that existing pre-pandemic differences might have lessened. Beyond examining overall rates and severity of mental health symptoms during the pandemic, it is important to establish the specific experiences of parents of children with rare neurogenetic conditions. This will enable us to understand factors central to parents’ mental health and ability to cope, which may differ from those reported in COVID-19 studies of parental wellbeing in the wider population.

To date, preliminary quantitative studies have begun to illustrate the impact of the pandemic on the mental health and wellbeing of families with rare neurogenetic conditions. However, qualitative approaches are needed as they facilitate the opportunity to access the thoughts and feelings of individuals, and how these are used to understand their experiences. Nonetheless the majority of qualitative research exploring family mental health and wellbeing in the context of the pandemic has focused on the experiences of mothers during the first COVID-19 wave (i.e., the first period of increased transmission, which in the UK is thought to be from March 2020 to September 2020 [8]). Moreover, research in families of children with SEND/ND has rarely specified the cause of SEND/ND [9–19].

Despite these limitations, in general, international qualitative findings suggest that during the pandemic children with SEND/ND experienced anxiety, low mood and social isolation, as well as difficulties understanding pandemic-related changes that manifested in increased challenging behaviour [9, 13, 15, 19]. These studies have also underscored the many challenges faced by parents of children with SEND/ND. Increased pressure on parents from the demands of concurrently working from home and providing childcare without typical support systems was commonly reported as negatively impacting parental mental health and wellbeing [9, 12, 14, 15, 18, 19]. Indeed, the majority of qualitiative research from the UK has focused on the effects of changes to the delivery (e.g., via telehealth systems) or removal of health, social and educational services for familes with SEND/ND. For example, surveys by Genetic Allicance UK and the Disabled Children’s Partnership [20, 21] describe how parents of individuals with rare conditions and disabilities reported feeling abandoned or left behind to cope with their children’s needs during the first UK lockdown, whilst becoming increasingly worried about long-term health implications. Moreover, these surveys highlight that despite some benefits (i.e., alleviating the burden of travelling to specialist clinics), alternatives such as telehealth appointments were implemented without assessing the needs or resources of the community generating inequity in their accessibility (e.g., for patients without access to technology, or communication and sensory difficulties). These findings might be particularly relevant to families of children with rare neurogenetic condtions for whom multidisciplanary services are essential.

However, as described above, the majority of qualitative studies have not specified the cause of SEND/ND, such that the specific challenges experienced by parents of children with rare neurogenetic conditions during the pandemic are not well known. Moveover, to our knowledge, the one study [7] which has examined the experiences of parents of children with a specified cause of ND only included mothers and was restricted to experiences in the first UK lockdown. This limits our ability to understand families’ experiences during subsequent restrictions and longer term impacts of the pandemic, wherein chronic difficulties and greater differences between families might have emerged. Therefore, it is important to establish what the specific challenges faced by families with rare neurogenetic conditions during the pandemic were by gathering detailed information via qualitative approaches, as well as identifying factors that fostered positive wellbeing. Consequently, in the current study we aimed to address how parents of children with rare neurogenetic conditions understand their experiences across the first year of the pandemic, the changes that occurred, the impact these changes had on their wellbeing, and that of their child with a rare neurogenetic condition, and how they coped with these changes across time. In doing so, we will be able to understand the lived experiences of families which have been overlooked by the majority of existing pandemic research.

## Method

We conducted semi-structured interviews as the qualitative component of a mixed-method research study—COVID-19 impact on wellbeing in families of children with rare neurogenetic disorders (CoIN) [6]—tracking the mental health wellbeing of children with rare neurogenetic conditions and their parents across the first year of the COVID-19 pandemic in the UK. Ethical approval was granted by the Psychiatry, Nursing and Midwifery Research Ethics Subcommittee, King’s College London (Ref: HR-19/20-18544).

### Participants’ eligibility criteria and recruitment

**Fig. 1:**
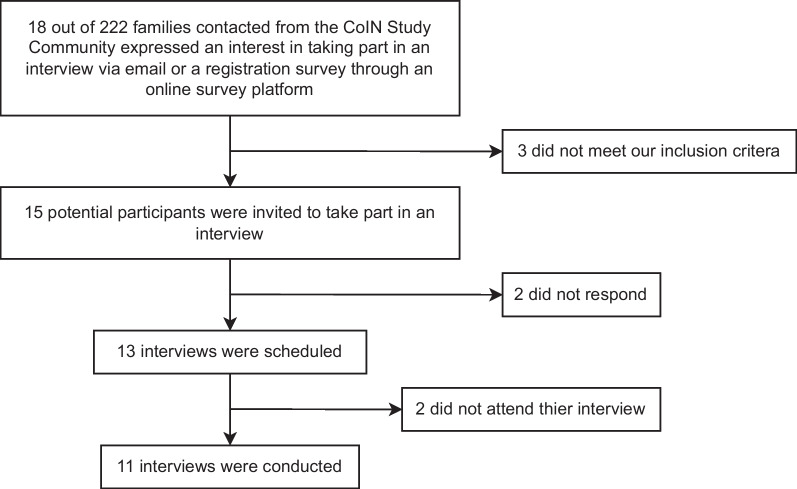
Recruitment procedure

Parents who had registered to take part in the CoIN study (*N* = 222) were invited to take part in this qualitative study via email. A total of 18 parents expressed an interest in taking part and were screened for inclusion criteria (parent of a child aged 0–15 years old with a rare neurogenetic condition—any rare neurological or neurodevelopmental diagnosis with a known or suspected genetic cause—and living in the UK) on a case-by-case basis, with assistance from a clinical geneticist (author KB). Diagnosis of a rare neurogenetic disorder was established by parent report. Of those eligible (*n* = 15), 11 parents provided written consent and completed an interview (8 mothers). Details of the recruitment procedure can be seen in Fig. 1. Participant characteristics are presented in Table 1.

**Table 1:** Participant characteristics

Participant ID^a^	Intellectual disability^b^	Condition type^c^	Child age (years)	Child Gender	Relationship to child	Parenting status
COIN3	Severe	Single gene disorder	3	Female	Father	Co-parent
COIN4	Moderate	Single gene disorder	15	Female	Mother	Co-parent
COIN5	Moderate	Descriptive disorder	9	Female	Mother	Co-parent
COIN6	Moderate	Single gene disorder	13	Male	Mother	Co-parent
COIN7	None	Multi-gene disorder	3	Female	Mother (adoptive)	Single parent
COIN8	Mild	Single gene disorder	11	Female	Father	Co-parent
COIN9	Severe	Descriptive disorder	12	Female	Father	Single parent
COIN10	Severe	Single gene disorder	8	Male	Mother	Co-parent
COIN11	Severe	Single gene disorder	10	Male	Mother	Co-parent
COIN12	Moderate	Other genetic disorder	4	Male	Mother	Co-parent
COIN13	Moderate	Single gene disorder	13	Male	Mother	Single parent

Conditions reported by parents included *DDX3*X-related neurodevelopmental disorder, *DYNC1H1*-related neurodevelopmental disorder, Coffin Siris syndrome, Sotos syndrome, Tatton-Brown-Rahman syndrome, PMM2-CDG, Leri-Weill dyschondrosteosis, Trisomy 9p and agenesis of corpus callosum

^a^Participant ID starts from COIN3 as ID was assigned when interviews were scheduled not after completion (see Fig. 1 for recruitment procedure). COIN1 and COIN2 did not attend their interviews

^b^Parent-reported level of disability. Each parent rated their perception of their child’s disability as none, mild, moderate or severe/profound

^c^Condition type was assigned with assistance from a clinical geneticist (author KB) as either a single gene disorder (Single Nucleotide Variant [SNV]), multi-gene disorder (Copy number variant [CNV] or multiple CNV), other genetic disorder (mitochondrial or chromosomal, including sex chromosome aneuploidy), or descriptive disorder (included those that are phenotypic)

### Procedure

Semi-structured interviews were conducted between 5th February and 19th March 2021, during the third national UK lockdown [22]. The interview schedule (Table 2) was developed from existing qualitative studies of parents of individuals with Intellectual Disability (ID) during the pandemic via communication with the authors [7, 16, 18, 23]. Building on these existing interview schedules, we added questions to examine the similarities and differences between parents’ experiences across pandemic phases, as a key aim of this research was to understand parents’ lived experiences beyond the first UK lockdown.

**Table 2:** Interview schedule (prompt/follow-up questions in italics)

**1. What was it like to normally care for your child (or insert name) before the outbreak of the coronavirus pandemic?**
*(a) What are the challenges and rewards?*
*(b) What services do you normally receive (medical, psychiatrics/psychological, behavioural, school/day services, respite)?*
*(c) What support do you normally get (friends/family)?*
*(d) Do you think others understand your caring responsibility and the impact it has on you and your family?*
**2. Thinking back to the initial lockdown in March 2020, what changed for you and your family?**
*(a) Daily life (i.e., routine for you and your child)*
*(b) Have there been any changes to the services you normally receive? Have any appointments been re-arranged, taken place online or been cancelled/postponed? How easy have you found accessing online/telephone appointments?*
*(c) Have there been any changes to the support (family/friends) you normally receive?*
*(d) Do you think others understand the impact of the pandemic on your caring responsibility and the impact it has on you and your family?*
**3. How has the coronavirus pandemic affected you?**
*(a) How do you think the lockdown/social distancing measures have impacted you (health/mood (mental wellbeing)/sleep/routines/relationships)?*
**4. How has the coronavirus pandemic affected your child?**
*(a) How do you think the lockdown/social distancing measures have impacted your child (health/mood (mental wellbeing)/sleep/routines/relationships)?*
**5. How has the coronavirus pandemic affected your family?**
*(a) How do you think the lockdown/social distancing measures have impacted other members of your family/household (health/mood (mental wellbeing)/sleep/routines/relationships)?*
*(b) How are you getting along as a family? / How is everyone getting along together?*
**6. Do you think you and your family’s experience of subsequent social distancing restrictions/lockdowns was similar to the initial lockdown in March 2020? If not, can you explain what changed?**
**7. What support have you/are you currently receiving?**
*(a) From family/friends/professional services*
*(b) How do you access support (i.e., what format—online/phone)?*
*(c) Is there anything that you don’t have at the moment that would help you manage better/In an ideal world, what resources or allowances would you need to manage lockdown/ to help your child?*
**8. What helps you most to cope with the current crisis? / Are there any coping strategies that you are using during this coronavirus pandemic? If so, what are these coping strategies and how have they helped you?**
*(a) Do you have any tips for other families in similar positions?*
**9. Have you experienced/Are there any positives from the pandemic? Are there any changes/have you learned anything that you are going to take forward for you/child/your family?**
**10. Looking ahead what are your main concerns/worries? Is there anything you are looking forward to (individual/child/family)?**
**11. Is there anything else you would like to add about your experience? / Is there anything else you would like to discuss that we haven’t already asked about in relation to your experience?**
**12. Do you have any questions for me?**
**13. Finally, what is the most important thing for you that we’ve discussed today?**

Practice interviews were conducted by the lead author (JM) with two parents, prior to completing interviews with participants, and feedback on interview technique was given by experienced authors (GS and CR). This was to ensure collection of high-quality data, which is essential for high-quality qualitative research [24].

At the beginning of each interview, the interviewer (JM) explained that the purpose of the study was to further understand the experiences of families in the CoIN community across the different stages of the pandemic, and flexibly explore issues that were important or specific to individual families. Interviews were conducted virtually via video conferencing software. In some instances (*n* = 6), interviews were observed by author KR. Interviews lasted 55 min on average (range 42–74 min). All interviews were audio and video recorded and were transcribed verbatim by author KR. Participants were thanked and reimbursed for their time. For a reflexivity statement see Additional file 1.

### Analysis

Interpretative Phenomenological Analysis (IPA) is a theoretical framework for qualitative inquiry which facilitates a detailed examination of personal lived experience, its personal meaning and interpretation [25]. IPA draws upon idiographic, hermeneutic, and phenomenological philosophical approaches to enable a case-by-case exploration of possible interpretations of how an individual makes sense of a salient experience (e.g., living through a pandemic) [24]. Commitment to these theoretical perspectives is achieved via the collection of detailed, descriptive and/or reflective data from small groups of participants selected based on shared perspectives (e.g., being a parent of a child with a rare neurogenetic condition), to achieve a degree of homogeneity [26]. IPA assumes that individuals interpret and assign meaning to their experiences and that researchers can reflexively re-interpret individuals’ sense-making, such that the analysis progresses from descriptive to interpretive, situating an individual case within more general claims.

We adopted IPA for this study above other qualitative approaches as it offered the best opportunity to explore the lived experiences of parents of children with rare neurogenetic conditions during the pandemic (for a detailed discussion of how IPA differs from other qualitative approaches see [27]). Data analysis was conducted manually as recommended for researchers new to IPA [24], and does not hinder creativity, unlike artificial frameworks in qualitative software packages [28]. Lead author (JM) flexibly employed the processes and principles of IPA outlined in Smith, Flowers and Larkin’s [24] guide for completing high quality IPA. Analysis steps are outlined in the Additional file 1.

## Results

We identified 4 key themes, each of which is discussed in relation to the shared and unique experiences of participants who endorsed them (Table 3).

**Table 3:** IPA analysis master themes and participants who endorsed them

Master theme	Participants who endorsed master theme
A varied impact on child wellbeing: from detrimental to ‘no big drama’	COIN3, COIN4, COIN5, COIN6, COIN7, COIN8, COIN9, COIN10, COIN11, COIN12, COIN13
Parental mental health and wellbeing: impact, changes and coping	COIN3, COIN4, COIN5, COIN6, COIN7, COIN8, COIN9, COIN10, COIN12, COIN13
‘The world had shut its doors and that was that’: care and social services during the pandemic	COIN3, COIN4, COIN6, COIN7, COIN8, COIN9, COIN10, COIN11, COIN12, COIN13
Time and luck: abstract concepts central to parents’ perspectives of how they coped during the pandemic	COIN3, COIN4, COIN5, COIN6, COIN7, COIN8, COIN9, COIN11, COIN12

Participant ID numbers are from 3 to 13. COIN1 and COIN2 are not valid participant IDs

### A varied impact on child wellbeing: from detrimental to ‘no big drama’

This theme addresses the times where parents spoke about the impact the pandemic had on their child with a rare neurogenetic condition’s mental health and wellbeing. In particular, the ways in which parents reflected on the reasons for their child’s emotions and behaviours during the pandemic, as well as how they as parents responded or coped with these changes.

A majority of parents expressed changes in their child’s emotions or behaviours during the pandemic, for example increases in anxiety-related (i.e., increased arousal) and autistic-like behaviours (i.e., restricted and repetitive behaviours, and stimming), as well as desire to understand why these changes might have occurred. This was rooted in parents’ role as their child’s primary caregiver and reflects their priority to support their child’s wellbeing. Initially, challenges understanding what the pandemic meant, in terms of risk of infection and COVID-19 measures, in addition to coping with constant changes in routines was, for many parents, key to their child’s emotions and behaviours during the pandemic. As a result, many parents hoped behaviours that were challenging to manage, including aggressive outbursts and meltdowns, would subside with the reinstatement of routine or the return to school. COIN10, for instance, described how their child was constantly confused about whether they themselves were ill, and why they could not go to school throughout the pandemic, but noticed some improvement with routine:

he just went crazy, really. He . . . couldn't cope with being in all the time, not doing the things he wanted to do, not having his routine of school . . . his behaviour was the main problem, and his confusion and feelings of worry himself that he must be ill, and… I think that fed through to behaviour.

At the same time, all parents expressed compassion when discussing changes in their child’s behaviours as many were cognisant of their own struggles managing uncertain and unpredictable pandemic measures. As COIN7 says:

I’ve noticed changes to her behaviour, and I think it’s been this up and down of suddenly, you can go to a shop, no you can’t, we’re back in lockdown. Or you can go to school . . . no you can’t we’re back in lockdown. . . . it's difficult enough as an adult to keep on top of it and figure out what you can do and understand why you can’t do things. Um, but there’s just no consistency, . . . and that’s been particularly hard for [daughter] because she just wants to be out. [laughs]

Parents also acknowledged the importance of social interactions for their child prior to the pandemic and were empathetic to the impact social restrictions had on their child’s wellbeing, as well as development. For some parents, over time this grew into worries about their child’s quality of life and whether they could support them. For example, a lack of social contact and an inability to independently use technology to socialise caused COIN5 to become concerned about her child’s shrinking social circle, whilst simultaneously struggling with the demands of being the only person who could support their child:

Um.. and it’s just, it’s been hard, I think she spends a lot of time with her grandparents, both sets, loves them to death, and just, hasn’t had that interaction with them. . . . my sister’s very good and will Facetime her, erm, once a week. Um, but again that… she can do that with an adult, but that’s hindered her in the fact that she hasn’t been able to do that with . . . her friends, because she needs an adult [to] bridge that gap for her . . . so, her social circle has got smaller and smaller, er, and then you become that, that one person or that one, one anchor and then, yo-, some days you don't wanna get up and play dolls or, or put things together, you just want to just, shut yourself away for 10 minutes, and just go… [mimed screaming] and you can't, because you're the only thing that she's got.

This extract highlights the tensions some parents experienced between the demands of caring for a child with a rare neurogenetic condition and their own wellbeing during the pandemic. We see that many parents were highly receptive to their child’s changing needs and emotional states, reflecting their priority to support their child’s wellbeing, sometimes even at the cost of their own mental state. For instance, COIN13 described how some of the most challenging times during the pandemic were when their son with a rare neurogenetic condition’s anxiety influenced them and their daughter:

I would say anxiety breeds anxiety because I can feel myself getting anxious when [son] gets anxious . . . like a pressure pot of anxiety through the whole house. Um and I think that was probably, the, the worst times.

However, not all parents reported that their child was negatively affected by the changes or restrictions bought on by the pandemic and for some parents the pandemic offered the opportunity to fully realise their child’s strengths and achievements. For example, COIN4 expressed pride regarding her child’s resourcefulness to develop her peer network via social media during the first UK lockdown and that COVID-19 restrictions had been ‘no drama’ for their child. Moreover, many children with rare neurogenetic conditions receive therapies or interventions, and in multiple cases parents worried whether their child would retain any developmental progress they had made. For instance, the fact that COIN3’s child retained skills after prolonged periods without intervention was a ‘surprise’ given they felt their child’s development had been ‘unpredictable’.

In the context of changes to typical routines and expectations of children, some parents also described gaining valuable insight into their child’s needs. COIN12, for instance, spoke of how the first UK lockdown truly benefited them, as they were able to spend more time as a family before their child started school. Additionally, more time enabled COIN12 to recognise how fixed their child’s behaviour and language was around everyday school and home routines, prompting them to be proactive in seeking support. Indeed, many parents suggested the pandemic offered the opportunity to reflect on their child’s needs, which appeared to be rooted in wanting the best for their child both now and in the future. For instance, time together made COIN8 question what was important in life for their daughter:

The thing I will always fall back on is that even during lockdown when she’s had her, her life experiences, er, reduced to basically staying at home, she’s still happy, she’s still content. So when she has more than this now, she’s likely to be happier, and I think that’s the thing I keep… falling back on. . . . there’s some things she’s missing out on because of her condition, but, stuff that really matters, I think she’s got, and that’s what COVID’s allowed us to foc- on, focus on is the stuff that actually really matters.

In summary, this theme illustrates the varied perceptions of parents regarding the impact of the pandemic their child’s wellbeing, and how parents responded to and reflected on their child’s emotions and behaviours. Understanding and supporting their child can be understood as central to parent’s pandemic experience and reflects a shared desire for wanting the best for their child.

### Parental mental health and wellbeing: impact, changes and coping

With new and increased demands from multiple roles (e.g., working from home and home schooling) alongside reduced support and higher uncertainty driven by the pandemic, multiple parents reflected upon their own mental health and wellbeing. Indeed, many parents expressed they tried to balance their work and personal life, but this was challenging. For instance, COIN6 described how this balancing act left them feeling divided and guilty about spending time on one, when the other might suffer. Moreover, COIN5, for example, spoke of how they became aware they were making many sacrifices to their wellbeing in order to balance expectations to work, as well as care for their children, that were not reciprocated by their partner, such that it not only impacted how they felt, but their relationship as well:

COIN5: really difficult, erm to kind of get that balance where, actually I was expected to work, . . . and all the other things that suddenly became part of my role as a teacher, that you know, wasn’t part of my role, but as well as trying to balance what was going on in the home as well.

JM: Um, how did that make you feel, in like your day to day?

COIN5: Stressed. Stressed, anxious, tired and cross, as well. It was, you know, it was one of those, where it was almost like "well my jobs more important than yours, so I'm just going to work mine, and you can do it [childcare], you'll just have to fit in with things". So, it just… yeah, it became…yeah, frustrating.

This extract from COIN5 draws our attention to the many demands that were placed on parents and how this became a central feature of how they felt during the pandemic. In addition, many parents described how their free time was infiltrated with domestic jobs they could not do whilst caring for their child during lockdowns. As COIN 10 says:

before the pandemic, um… you know I’d have time during the day to get on with everything that I need to be doing. And… that kind of totally went. So, you know, obviously there was no free time at all, all my time… was… with the kids and with [son with rare neurogenetic condition], you know there’s no let up. You’re trying to do an online food order and he’s there climbing all over you. . . . So yeah, I mean it just got exhausting, I have no time to read a book or just do anything for any downtime.

Not having any personal time and limited social contact appeared to be especially challenging for single parents, who described limited or no opportunities for in-person support during the pandemic As COIN7 says:

I did sign up to have three kids, but I didn't sign up to it in a pandemic where my entire support systems taken away. Um… and you’re just on your own [laughs] and it was just a real, shock to the system

Despite experiencing many difficulties during the pandemic, few parents reported utilising negative coping strategies. Instead, many sought positive experiences and reported factors that buffered against the impact of the pandemic on their wellbeing. For example, some parents acknowledged the importance of doing something for themselves, and being able to express their opinions and feelings either to a friend or mental health professional. Other parents reported more quality time together and family activities, as well as the flexibility of remote working as positive experiences they wanted to retain beyond the pandemic. However, it was not possible for all parents to negate the effects of the pandemic on their wellbeing and many factors may have prevented parents from seeking social and emotional support. For example, COIN13 described loneliness and worrying about emotionally burdening others:

Just felt really lonely . . . everything going on in my head, and sometimes you just need to talk about it and get it out of your head, and I felt like I had lost that, I'd lost that, um… ability to do that. And I think because everybody was going through such a terrible time, and everybody felt anxious about everything. I kind of felt like well I can't dump all of my emotions on anybody else anyway.

### ‘The world had shut its doors and that was that’: care and social services during the pandemic

Given that many children with rare neurogenetic conditions in the UK require multidisciplinary care, it is unsurprising that the closure of healthcare and social services, as a result of COVID-19 measures, was highly relevant to the experiences of parents during the pandemic.

Initially, parents reported welcoming respite from healthcare services. However, this was short-lived as the laborious experience of managing and attending multiple appointments, as well as advocating for care prior to the pandemic intensified with the new demands imposed on the UK’s healthcare system. Parents described how they were expected to attend both in-person and telehealth appointments, organise PPE, and liaise with an unfamiliar and reduced workforce. Indeed, for many parents this experience felt like a ‘nightmare’. Moreover, an exacerbated sense of being solely responsible for their child’s medical care seemed like a common occurrence. As COIN6 explains:

So y-you kind of ended up having to be, er even more so I guess than, than pre-COVD, sort of, I guess, the person that, that, erm, maintained the status quo and was able to kind of ferry information out to everybody. Er, so, you, end up becoming [laughs] sort of a bit of a care coordinator

Parents also expressed concerns over the appropriateness and effectiveness of alternative service provisions (i.e., telehealth appointments). For example, COIN10’s child’s school offered to provide physiotherapy over Zoom, but they felt this was inappropriate as their son struggled to cooperate online and with them, as a parent, delivering physiotherapy at home, which was deemed schoolwork by their child. COIN13 adds to this feeling of ineffectiveness by describing how they struggled to engage with or get anything from online appointments during the pandemic:

with [son’s] anxiety just, um… trying to explain to someone over the phone, um… was… was very difficult because [son] was there . . . I felt again like I can’t really say [how home life was] because . . . the people who it’s really tough and difficult for . . . could potentially be listening . . . if felt a bit like floundering . . . the appointments were just touching the edges . . . another appointment. . . that was really difficult [was physiotherapy]. . . because you know trying to put a camera around to show, um,… what you think the problem is… she [therapist] couldn’t pick it up properly. Um, so I think it’s difficult from both perspectives, you know from parent… the patient and the practitioner. I think it’s just really, um, it’s just not a great scenario.

The extract from COIN13 illustrates simultaneous challenges getting care needs met on the one hand, and empathy for healthcare providers on the other. Indeed, other parents appreciated individual professionals were doing the best they could in circumstances which no one wanted, such that it was not worth blaming anyone and better to keep a level head. These ways of thinking might have manifested from past experiences of advocating care, which some parents felt was impossible even prior to the pandemic, such that compliance with the situation might have buffered against the potential negative impact on parents’ wellbeing. Acceptance is hinted at in this extract from COIN7:

Um, see I don’t have any issues with how they’re doing it because there’s no choice in the situation we’re in, but I do think there’s going to be an impact on the kids, I do think that… they would make more progress… with more support, um particularly [daughter], you know, managing pain is a huge issue cause no one really understands it properly. Um… but yeah, again, just is what it is.

However, with time parents acknowledged that they became increasingly anxious and concerned about the practical repercussions of being unable to access medical interventions and equipment. Indeed, many parents felt responsible for mitigating the unknown impact of the pandemic on long-term health and psychological outcomes. For example, COIN12 described how the uncertainty of the impact of losing services was their biggest worry during the pandemic, and that despite paying for any private therapy they could, there were some services their child was still unable to access. COIN12’s experience highlights the emotional and financial inequity of care during the pandemic:

it felt like it wasn’t fair, and it feels like he gets a bit lost in the system sometimes because he is so complex, that they just… they don’t know what to do for him . . . And you feel kind of like they wash their hands a little bit of it, which I know isn’t true, but that’s how it makes you feel . . . when you see other people getting that [therapy] and you don’t get that. And yeah, it can be really frustrating and the struggle to get him what he needs… is pretty exhausting cause you’re constantly fighting, constantly trying to get… to get what he needs in place. And yeah, that can take a lot of effort.

While COIN12 expressed feelings of frustration and how they battled to get their child’s care needs met, other parents were hesitant to burden a fragile system or unsure what support their child needed without the input of professionals. COIN11 adds to this as they reflected on whether changes to healthcare and other services, as a result of COVID-19 measures, affected how they felt about their child’s condition. This extract highlights how some parents’ worries did not change and matched pre-pandemic anxieties, which appeared to be rooted in a lack of knowledge surrounding their child’s rare condition:

Ah… well it’s really stressful, but also it’s always stressful with [son] because we never know what's going to happen with him. I mean, because he's such, he's got such a rare disorder that nobody really knows how it's going to go, so it's, it's… it causes anxiety, but not anymore than it would…anyway. There’s always a level of anxiety that’s there, it’s not gonna, yeah, it’s gonna make it massively different. Um, but yeah, it is stressful to think that things aren’t being monitored, that is a worry.

Multiple parents also reflected on their hopes and wishes that people would learn how to better support the diverse needs of children with rare neurogenetic conditions and their parents by learning from the pandemic. As COIN6 says:

I think there, you know there does need to be… [sighs] some time to kind of reflect and take stock of… how… things have sort of impacted, yes, a diverse group of people . . . I'd hoped that there's some things and some lessons that people can take away from it in thinking about how to support families and services.

Similarly, COIN8 said:

I think kids with disabilities is an area that, you know, it’s not a luxury, it’s not something, um, that, um, people chose to have, and I think as a country we should be far more supportive, um, especially families where it’s needed more, that kind of proportional, erm, support I think’s, er, is crucial. Um, I really, really like to see as a consequence of this, cause I suspect if it’s not this current version of COVID there may be some mutation that comes around every year, we get resilient services to support families that are most in need, but it’s not just… speech and language, or physio, or, or, or paediatricians, it’s financial support for families as well.

### Time and luck: abstract concepts central to parents’ perspectives of how they coped during the pandemic

All parents reflected on how they had coped during the pandemic. Here, we explore how the passing of time and luck were central to many parents understanding of how they coped.

During the first UK lockdown, some parents were fearful of what would happen to their child if they caught COVID-19, which appeared to be rooted in previous experiences of receiving limited clinical information about their child’s rare condition, or the consequences for their child if they, as their primary caregiver, were to be infected. Over time, this fear diminished as COVID-19’s virulence was better understood, and parents practiced infection-control measures, such that social distancing became the ‘new normal’. COIN11, for example, spoke of how during the first UK lockdown the pandemic felt apocalyptic, whereas at the time of the interview (i.e., during the third UK lockdown) they felt more at ease:

we were really terrified, like beyond terror, it was like the zombies were coming to get us and coronavirus was slowly infecting people closer and closer, so we didn't go out the house at all because, um, we didn't know anything about it, and we were quite convinced that if [son] was to catch it, it was going to kill him. . . . now we’re a lot more relaxed about it, but… the first one we were really, really strict.

Similarly below, COIN7 described how it was difficult to cope with changes brought on by the pandemic as a single-parent, but with time they were able to manage:

there’s been points where I’ve found myself anxious, um, or really stressed out and feel like I can’t do everything. Again, things like appointments, I haven’t got my usual support network. Um, so it’s a really difficult balancing action, but again it feels like things are getting easier… um as times gone on

The extract from COIN 11 and COIN7, draw our attention to the importance of the passing of time for how parents felt they were able cope. Moreover, parents expressed they built-up resilience by dealing with the unique experiences of being a parent of a child with a rare neurogenetic condition during the first UK lockdown, and subsequently felt more adaptable and self-sufficient across phases of the pandemic. The metaphor which COIN4 used below functions as a direct *sense-making* response and communicates how the passing of time felt for many parents:

its' like putting on it might be uncomfortable shoes, but you know how they're gonna feel [laugh]

Although we have indicated that the metaphor above could reflect many parents’ descriptions of becoming more resilient and adaptable to the challenges thrown at them by the pandemic, it does not necessarily mean that with time parents found it easier to cope, especially when individuals with disabilities and their families were often perceived as an afterthought by policy makers. For example, the easing of lockdown restriction in January 2021, led COIN12 to realise that they had found it easier to cope with more severe pandemic restrictions than they had initially thought. Moreover, a few parents reported feeling less optimistic and positive as time went on, which appeared to be rooted in earlier experiences of the UK government’s false promises of an end to pandemic restrictions. AS COIN5 says:

horrible as it is, I'd rather just keep going at it, and then when we come out of it, hopefully that will be… the point where we can move forward with it. But if we come out too soon, we'll just be right back in it again. And I, I just, that would send me into the urgh, no, I can't.

While it appeared time could be central to how some parents reflected on their ability to cope across the pandemic, for other parents luck was an important concept. Despite challenges inherent to being a parent of a child with a rare neurogenetic condition, a few parents expressed that they felt fortunate for their position when they explored how they felt they had coped relative to other families, with or without children with rare neurogenetic conditions. As COIN9 says:

The pandemic has not affected us in the way I would say it probably has affected… 99% of people you know so where . . . [it] sounds… strange and odd, but probably because of [daughter’s] disabilities and me being her full time carer, we're, we're some of the lucky ones, um, because we've got a good, um… support network around us

Similarly, below COIN8 reflects on their experiences compared to other families, highlighting how some parents perceived luck and good fortune as central to how they coped during the pandemic:

I spend a lot of time thinking about, how lucky we are, how fortunate we are. So whilst [daughter has a rare neurogenetic condition] . . . reflecting on the changes everyone’s had to go through we’re lucky we’ve come through this relatively unscathed and actually with some positives, rather than some families where, they’ve lost pretty much everything, you know, they’ve lost their income, lost their stable housing, they’ve lost all external supports that maybe they were relying on.

## Discussion

The current study explored the experiences of 11 parents of children with rare neurogenetic conditions, who were living in the UK during the first year of the COVID-19 pandemic (March 2020–March 2021). To our knowledge, it is the first qualitative study of both mothers’ and fathers’ experiences, and of these families’ experiences beyond the first UK COVID-19 wave. Across this period, many parents faced changes in their child’s behaviour (e.g., increased challenging behaviour), which they perceived as their child’s response to disruptions in their typical routines, or ability to understand pandemic measures. Fewer parents reported positive impacts on their child’s wellbeing. The pandemic context also offered many parents the opportunity to reflect on their children’s abilities—their currents strengths and support needs, as well as their future. Moreover, parents reported that caring for a child with complex needs can be mentally draining, and that this was exacerbated by increased demands such as working from home, and a lack of timely, effective, and appropriate support or respite. Despite these challenges, families reported positives as well as resilience and adjustment over time.

In line with other COVID-19 related studies on the mental health and wellbeing of children with rare neurogenetic conditions [6, 7], children with SEND/ND without a specified cause [9, 13, 15, 17, 19, 29, 30], and children in the wider population [31, 32], parents in the present study reported changes in their child’s emotions and behaviours during the pandemic. For example, consistent with previous reports [7, 9, 13, 15, 19, 32], many parents perceived increases in anxiety-related and autistic-like behaviours in their child as a response to pandemic measures, and were concerned about the effects of their child being unable to access technology for socialising. Many parents in the present study also reported feeling responsible for understanding the root of their child’s difficulties and implementing support, which was embedded in wanting the best for their child now and in the future. However, this sometimes came at the cost of parents’ own needs and wellbeing. Moreover, parents’ desires to best care for their child was conflicted with increased demands to do so via the withdrawal of pre-existing support structures, and pressures to balance other responsibilities (i.e., working from home). Tensions between childcare and work responsibilities are echoed in qualitative studies of parents in the wider population [33, 34], which have described parents’ concerns about being unable to meet their child’s needs during the pandemic. Moreover, a lack of information, guidance, and social-emotional support has been identified as key to how parents with rare neurogenetic condition coped during the pandemic [7].

Despite similarities between the pandemic experiences of parents reported in the current study and studies of parents of children with and without SEND/ND, differences in the needs of these groups of children are reflected in the worries of parents. In the current study, altered and terminated healthcare provision was reported as central to parents’ pandemic experience and ability to cope, whereas home schooling demands have been commonly reported and focused on in studies of parents of children in the wider population and with ND without a specified cause [33–35]. In particular, parents in the current study reported increased pressure to serve as a care coordinator, and concerns regarding the appropriateness and effectiveness of alternative service provision. These have both been highlighted in other pandemic reports of individuals with rare conditions [20, 36–39]. Moreover, our study revealed the significant emotional impact of closing healthcare and social services on parents which in other studies has been associated with parental feelings of abandonment [7, 18].

Further, it is notable that the experiences described by parents in the current study are strikingly similar to those reported in studies of families with rare neurogenetic conditions prior to the pandemic. For example, pre-pandemic studies have reported that parents of children with rare neurogenetic conditions feel responsible for managing their child’s condition via fulfilling a care coordinator’s role [39] and support their child’s wellbeing and development with a lack of guidance and mental health support [36, 40]. Both of these challenges were decribed by parents in the current study. As such, the pandemic might have served as a context in which the trials and tribulations of daily life for families of children with rare neurogenetic conditions have been brought into sharp focus [41]. Consequently, researchers should work closely with policy makers to implement programs which enable parents to support their own wellbeing and their child’s development, such as group-based mindfulness programs [42] or the Early Positive Approaches to Support (E-PAtS) intervention [43, 44], in future emergency and non-pandemic contexts.

Despite multiple challenges, parents reported some positives, including time to focus on their child’s skills and needs without educational and societal pressures, and more quality time as a family. Parents also described going through a period of adjustment and developing resilience to deal with future challenges. Nevertheless, for some parents positives were limited to periods of heightened restrictions (i.e., national lockdowns). Factors which influencing coping and adjustment during the pandemic warrant future investigation in order to identify parents most at risk of persistent negative impacts of the pandemic.

The results of the current study should be interpreted in light of several limitations. First, our group of participants was relatively large for IPA research. Smith and colleagues [24] posit that a group of three participants is optimal for researchers to meet the commitments of IPA without being overwhelmed by the amount of data generated from first person interviews. In particular, Smith and colleagues [24] suggest that analyses with larger groups of participants tend to de-emphasise the detailed examination of each case, and instead focus on what is recurrent and more general across cases. Despite, the challenges of applying IPA to a larger group of participants, we were still able to examine meaningful similarities and differences in parents understanding of their pandemic experience.

Furthermore, IPA research calls for the use of highly homogeneous groups of participants, yet we included parents of children with multiple rare neurogenetic conditions and across a wide age range. As a result, there might be important differences that need to be considered. For instance, COIN9’s child was severely disabled, but for them this was not a complicating factor during the pandemic as they retained support. This was not true for all families and care needs might have impacted the experiences of parents. Moreover, condition-specific pandemic experiences might exist. For example, preliminary evidence suggests there might be subtle differences in the magnitude and type of responses to the pandemic between subgroups of children, such as those with SEND/ND [6, 31, 45] or mental health difficulties [46]. Additionally, the impact of the pandemic might be more persistent for children with specific diagnoses, for example, Asbury and Toseeb [10] found heightened worry remained more stable over the first 6-months of the pandemic for autistic children than those with other SEND/ND.

In addition to the challenges to the commitments of IPA described above, our interview schedule was long, extensive and detailed, as we were unsure which elements of family life and wellbeing would be the most important to parents. Consequently, our interview schedule covered a wide range of topics consistent with qualitative studies exploring experiences of families with ID during the first UK lockdown [7, 16, 18, 23], which may have influenced our understanding of parents experiences.

Further, participants in this study were predominantly white, relatively educated and affluent. However, unlike previous studies of families of children with rare neurogenetic conditions during the pandemic [7] we included both mothers and fathers. To build on this, future studies could adopt a multi-perspective approach by including other family members, professionals, and children with rare neurogenetic conditions themselves. This approach has been used successfully in qualitative studies of families in the wider population during the pandemic [32, 34] and would facilitate a holistic view of families experiences across the COVID-19 pandemic.

## Conclusion

Consistent with findings from quantitative studies of children with rare neurogenetic disorders during the pandemic, parents in the current study perceived the pandemic as negatively impacting their child’s behaviour and wellbeing. By using IPA, we were also able to identify how parents interpreted and responded to the impact of the pandemic on their child. Moreover these findings provide unique insights into the impact of changes to multidisciplinary services on families, as well underscoring the day-to-day challenges experienced by parents. We now have the opportunity to address these challenges in a non-pandemic context by exploring factors that influence coping, and developing appropriate and useful information, guidance, support services and policies.

## Supplementary Information

**Additional file 1**. **Supplementary Material:** Reflexivity Statement, IPA Analysis Steps, and CoIN Study group.  

## Data Availability

The data that support the findings of this study are not publically available as they contain information that could compromise the participants’ privacy. Inquiries about the qualitative materials used in the manuscript can be made to the corresponding author.

## Abbreviations

COVID-19: Coronavirus disease 2019
UK: United Kingdom
CoIN study: COVID-19 impact on wellbeing in families of children with rare neurogenetic disorders study
SEND/ND: Special educational needs and disability or neurodevelopmental disorder
ID: Intellectual disability
IPA: Interpretative phenomenological analysis
Co-SPACE study: COVID-19: supporting parents, adolescents and children during epidemics study
