# Supplementary material for: Experiences of parents of children with rare neurogenetic conditions during the COVID-19 pandemic: an interpretative phenomenological analysis
Source: BMC Psychol. 2023 Jun 8;11:179. doi: 10.1186/s40359-023-01205-3 (PMC10249551; doi:10.1186/s40359-023-01205-3)
Supplement: Supplementary file 1 — Additional file 1. Supplementary Material: Reflexivity Statement, IPA Analysis Steps, and CoIN Study group. [file 40359_2023_1205_MOESM1_ESM.docx]

**Experiences of parents of children with rare neurogenetic conditions during the COVID-19 pandemic: an interpretative phenomenological analysis, Additional File 1.**

**Supplementary Information**

###

**Reflexivity Statement**

Reflexivity is an essential component of qualitative research and involves ‘self-monitoring of, and a self-responding to, our thoughts, feelings and actions’ (1, p. 337).

**Prior assumptions and experience**

Authors who were involved in the collection, pre-processing and analysis of data, needed to take in to account the ways in which their interactions with participants and participants’ data could be shaped by their own professional and personal background, experience, and prior assumptions. The two researchers (JM and KR) who had face-to-face contact with the participants during their interviews, which were conducted remotely using video conferencing software, were both early career researchers in psychology. The interviews were conducted by JM and over half were observed by KR. Verbatim transcription was completed by KR. IPA analysis was completed by JM. During data collection and transcription, JM was a research assistant and KR was an honorary research assistant. Whilst analysing the data and preparing the manuscript, JM was a PhD student in medical sciences and KR was a graduate in psychology.

**Relationship with Participants**

No members of the research team had a prior relationship with participants.

**IPA Analysis Steps**

Lead author (JM) flexibly employed the processes and principles of IPA outlined in Smith, Flowers and Larkin’s (2) guide for completing high quality IPA. Analysis steps are outlined below:

1. **Reading and re-reading:** familiarisation with interview content, by watching recordings and reading transcripts. Initial thoughts were noted down separately and addressed in the next step.
2. **Initial noting:** the semantic content and use of language was explored using three levels of comments: (1) descriptive comments described the content of what the participant had said (i.e., things that mattered to the participant – key objects, events and experiences); (2) linguistic comments explored the use of language by the participant (e.g., pauses, laughter, tone, use of metaphors); and (3) conceptual comments examined abstract concepts which make sense of participants understanding of what they have described in the interview (e.g., if the participants descriptions of their experiences were embedded in time, it could suggest they have come to terms with it now, or they experienced periods where they were more vulnerable). These comments were written next to the transcript.
3. **Developing emergent themes:** initial notes were summarised into pithy statements in the context of discrete sections of the interview and the interview as a whole, and commented on the transcript alongside initial notes.
4. **Searching for connections across emergent themes:** a chronological list of emergent themes was generated and eyeballed to generate clusters using multiple grouping techniques (see (2)). Each cluster was given a gestalt title and then evidence from the transcript was extracted for each emergent theme within the cluster (‘superordinate theme’).
5. **Moving to the next case:** a break was taken between analysis of each transcript to facilitate separation between cases and the emergence of novel themes.
6. **Looking for patterns across cases:** a list of superordinate themes was generated for each case and examined for commonalities, by exploring how themes echoed and resonated across cases. An inductive approach to the examination of patterns, in which researchers hypothesised themes and revisited how they were experienced by each case, was adopted to increase the validity of theme generation across cases.

**CoIN Study group**

Michael Absoud^1^, Kate Baker^2^, Ted Barker^3^, Patrick Bolton^4^, Sarah Charles^3^, Hayley Crawford^5^, Emily Farran^6^, Megan Freeth^7^, Emily Jones^8^, Jessica Martin^3,2^, Jo Moss^6^, Sinead Rhodes^9^, Deborah Riby^10^, Caroline Richards^11^, Kathryn Robertson^3^, Abigail Runicles^3^, Gaia Scerif^12^, Emily Simonoff^4^, David Skuse^13^, Jo Traver^14^, Charlotte Tye^3^, Jo van Herwegen^15^, Jane Waite^14^, and Jeanne Wolstencroft^13^.

^1^ Department of Women and Children’s Health, King’s College, London, United Kingdom

^2^ MRC Cognition and Brain Sciences Unit, University of Cambridge, United Kingdom

^3^ Department of Psychology, Institute of Psychiatry, Psychology & Neuroscience, King’s College, London, United Kingdom

^4^ Department of Child and Adolescent Psychiatry, Institute of Psychiatry, Psychology & Neuroscience, King’s College, London, United Kingdom

^5^ Warwick Medical School, University of Warwick, United Kingdom

^6^ School of Psychology, University of Surrey, United Kingdom

^7^ Department of Psychology, University of Sheffield, United Kingdom

^8^ Department of Psychological Sciences, Birkbeck University of London, United Kingdom

^9^ Centre for Clinical Brain Sciences, The University of Edinburgh, United Kingdom

^10^ Department of Psychology, Durham University, United Kingdom

^11^ Cerebra Centre for Neurodevelopmental Disorders, School of Psychology, University of Birmingham, United Kingdom

^12^ Department of Experimental Psychology, University of Oxford, United Kingdom

^13^ Institute of Child Health, University College London, United Kingdom

^14^ School of Psychology, Aston University, United Kingdom

^15^ Institute of Education, University College London, United Kingdom

**References**

1. Corlett S, Mavin S. Reflexivity and Researcher Positionality. In: The Sage Handbook of Qualitative Business and Management Research Methods: History and Traditions. 2018. p. 377–98.

2. Smith JA, Flowers P, Larkin M. Interpretative Phenomenological Analysis: Theory, Method and Research. Th: SAGE Publications Limited; 2009.
